# The Louse Problem at the Front

**Published:** 1918-01

**Authors:** 


**The Louse Problem at the Front.** By A. D. PEACOCK, R. A. M. C. (T. F.), formerly Entomologist to the Government of Nigeria. Abs. from the *Brit. Med. Jour.*, May 27 and June 3, 1916.

P. first studies in detail the appearance, anatomy, and habits of the *Pediculus humanus* of the order *Anoplura* or *Siphunculata*. A number of microscopic drawings are presented. He finds no essential difference between black and gray lice. He notes particularly the strength of the legs and the sharp claws and spines, also the absence of any structures, except the eyes and antennae, that denote special senses. The insect feeds by sucking human blood for a maximum of 20 minutes twice a day. In the female 8 ovaries have been noted and it appears to lay 5 eggs at a time and to have a total capacity of 125 eggs. In experiments there has been a surprisingly large mortality among the young. The length of life is from 7 to 8 weeks. Vitality depends upon three factors : abundance of food, the normal body temperature, and body emanations. It appears that body moisture is the chief essential : but whether the humidity itself or its chemical quality is the favoring condition, is not known. In experiments, the louse could be maintained away from the human body for nearly nine days, but after the second day it became moribund.

As to the *habits* of the louse, P. finds that it depends upon clothing worn next to the body, and that it prefers the comfort of the seams. It is most numerous on the shirt, but most eggs are found at the fork of the trousers. Eggs are also laid in the armpits, the triangles at the tail of the shirt, the seams of both trousers and shirts, and the neck. There is also a general distribution of eggs. In cases badly infested they have been found on rosaries and on the flap seams of the pockets.

The maximum time that eggs may remain dormant away from the body is found by Warburton to be 40 days. At the front it has been found that they may survive freezing. It is therefore important to note " that eggs on the clothing, particularly the outer garments, if not treated regularly by ironing or disinfection, are a possible source of infestation for as long as a month after laying. Also, the removal of the clothing from the body for a few days in order to kill the eggs and lice by exposure, is not a practicable scheme. "

Although the louse's powers of endurance appear small, they are by no means inconsiderable. It " *is a parasite which is utterly dependent upon man's blood for sustenance and man's body and clothing for prolonged, prosperous longevity and reproduction* ".

As a result of experiments to determine the migratory habits of the louse, P. concludes that they migrate, but only in times of stress. He notes especially their habits of " congregating ". If left on discarded clothing, they invariably collect at its highest point because of the warmth, but show no disposition to leave. Light is not a stimulus to congregation. This characteristic of collecting indicates a gregarious habit and comfortable inertia, but it shows that competition is no incentive to migration.

Lice may wander from the host. It appears, however, that they do so only when the surroundings of the host are warm and comfortable, as in a bed, or where soldiers are crowded together. Thus infection may be carried. It is also clear that the lice find a host sleeping on infested bedding. This discovery may be due (1) to the warming of the bed, (2) to the contact of the lice with the body, (3) to the insect's " scenting ". It is impossible to tell which of these motives is most important, for no deliberate attempt to reach the host has been observed. The instinct of pursuit seems feeble, and there is probably little inclination to choose hosts. Two types suffer most from lice : the person with a sensitive skin, and the person who has never been infested before. Uncleanliness from service or from habit may cause a person to be badly infested.

Lousiness is felt mostly at night, probably for psychological reasons. It is an exceedingly distressing condition, causing serious loss of sleep and consequently impaired vitality. Scratching often leads to sepsis and the " louse rash " that may be taken for the scabies. These are the chief results on the western front; although the menace of a typhus epidemic from this source is also present but to a less extent than on the eastern front.

The author examined the clothing of about 300 men to determine the average number of lice and their distribution. He found an average per undershirt of 10.7 and an average per trousers of 4.2. The men were taken from 10 different units. 95 of the men examined were infested. Even when clean shirts were issued, at the end of two days the average amount of infesting took place. Reinfestation comes partly from eggs hatched in the trousers, partly from outer garments. In a few cases where groups of men changed and bathed weekly and were cleanly in keeping their billet, the average fell to 3.5 per man. There was about 5 o/o of very bad cases averaging from 168 lice per man to over 10,000. There appears to be about an equal amount of infesting in the open trench and in the dug-outs. Although these last abodes have the reputation of being the homes of lousiness, the writer after the most careful search was unable to find a single louse in one that was considered especially bad. Although he and an assistant slept in it two nights after the infested men had left, not more than one louse could be discovered on their clothing. 13 such dug-outs were examined with similar results. It appears also from a careful series of experiments that blankets, straw, and paillasses are only secondary sources of infestation. P. believes therefore, that the men themselves are the chief source, and that the conditions of proximity, especially in dug-outs, is the chief method of spread and propagation. He believes that their introduction into the army was due to uncleanly recruits, and that the spread is still largely the result of notably filthy carriers, a class of the soldiery that amounts to about 5 o/o.

Methods of Prevention and Destruction. — N. C. I. (naphthaline 96 o/o, creosote 2 o/o, and iodoform 2 o/o) has proved experimentally and in practice the best powder preparation. Its killing power is high and it is equally effective preventively. Too free a use of it at the fork, however, is likely to cause smarting.

Vermijelli ointment is very effective, but must be used to anoint the body from neck to knees. Otherwise it only causes the vermin to migrate from one part of the body to another.

Crude oil ointment (2 lb. of soft paraffin melted and 4 oz. of crude tar oil added) was used in the same manner and gave excellent results, showing a higher killing power than Vermijelli. Its efficiency as a deterrent was 97.7 o/o.

The use of N. C. I. powder on the shirt and trousers and of Vermijelli ointment smeared on the fork of the trousers and on the seams, resulted in a highly increased efficiency. Similarly the combination of N. C. I. and crude oil ointment was effective.

The author finds mercury ointment, white mercury powder, and sulphur, either impracticable or useless.

*Treatment of Infected Underclothing.* — In Thresh disinfectors the author finds that steaming at 215° F. for three quarters of an hour, or at 220° for half an hour is necessary. Boiling from 1 1/2 to 5 minutes is sufficient to kill eggs. 1 1/2 o/o cresol solution killed lice after an hour's soaking of garments, and also eggs. Similarly a 7 o/o chloride of lime solution used for 24 hours is effective; but alum has proved a complete failure.

Bathing and change of garments are of little value unless the outer garments are also disinfected. The only practical way of doing this is by ironing with well heated irons. This presents considerable practical difficulty, for it requires one ironer for each of a party of bathers passing through the baths in fifteen minutes.

The author appends various *recommendations for a practical campaign* against the pest. There should be provision for all necessary inspection and research. Sanitary officers should be organized and informed as to the best methods of procedure. Similarly there should be lectures and demonstrations for the men, and a pamphlet issued giving all necessary information as to prevention and treatment. The author urges every possible provision for bathing, change of clothing, and ironing. Most of this work must, of course, be accomplished while the men are out of the trenches, but they should be provided with all possible means of combatting the pest while they are on active duty.

